# Ocular Surface Squamous Neoplasia Masquerading as Superior Limbic Keratoconjunctivitis

**DOI:** 10.4103/0974-9233.75895

**Published:** 2011

**Authors:** Majid Moshirfar, Yousuf M. Khalifa, Annie Kuo, Don Davis, Nick Mamalis

**Affiliations:** Department of Ophthalmology and Visual Sciences, John A. Moran Eye Center, University of Utah, Salt Lake City, UT, USA

**Keywords:** Conjunctival Intraepithelial Neoplasia, Masquerade, Ocular Surface Squamous Neoplasia, Superior Limbic Keratoconjunctivitis

## Abstract

To report a case of ocular surface squamous neoplasia (OSSN) masquerading as superior limbic keratoconjunctivitis (SLK). A 62-year-old woman was referred with foreign body sensation, irritation, photophobia and decreased vision in the left eye. She was initially treated for 10 months with intermittent topical corticosteroids for a presumed diagnosis of SLK. She underwent excisional biopsy of the superior conjunctiva and was found, on histopathologic evaluation, to have OSSN with moderate to marked dysplasia. This is the first reported case of OSSN masquerading with signs and symptoms of SLK. Any ocular surface lesion refractory to standard medical treatment should raise suspicion for a malignant process and warrant further cytologic or histopathologic evaluation.

## INTRODUCTION

Conjunctival ocular surface squamous neoplasia (OSSN) lesions are described as papilliform, leukoplakic or gelatinous, appearing mostly at the limbus. These lesions are considered *in situ* if restricted within the epithelium and may be termed conjunctival intraepithelial neoplasm or CIN. On the other hand, superior limbic keratoconjunctivitis (SLK) presents as chronic superior bulbar conjunctival injection and thickening, believed to be associated with mechanical soft tissue microtrauma. In either case, patients can present with similar symptoms, including foreign body sensation, irritation and redness.

OSSN has been reported to present as corneal ulcer,^1^ chronic blepharoconjunctivitis,^2^ pterygium,^3^ necrotizing scleritis^4^ and sclerokeratitis.^5^ This is the first reported case of OSSN masquerading as SLK.

## CASE REPORT

A 62-year-old woman was referred with chronic redness, irritation and photophobia of the left eye for several months. She had no prior history of eyelid surgery or trauma. Her past medical history was significant for hypothyroidism. Multiple treatment regimens, including artificial tears, topical corticosteroids and bandage contact lens, had been tried. On examination, the best-corrected visual acuity was 20/20 in both eyes. Her left superior bulbar conjunctiva was injected with engorged and tortuous episcleral vessels [Figure 1]. In addition, there was a single filament present on the superior corneal surface but no epithelial leukoplakia was noted. The diagnosis was felt to be SLK on clinical grounds and treatment with fluorometholone 0.1% ophthalmic ointment four-times daily was started. At the 3-week follow-up, her symptoms improved and the superior limbus and conjunctiva had not changed. Prednisolone acetate 1% eyedrops three-times daily was added and, 1 month later, she reported resolution of her symptoms and had decreased superior conjunctival injection. Over the next 3 months, she was treated for sporadic recurrence of her symptoms with tapering doses of corticosteroid drops and ointment. Within a few weeks of tapering the topical corticosteroids, she had recurrence of her symptoms. At this point, the superior bulbar conjunctival lesion developed an elevated, fibrinous appearance that extended onto the corneal epithelium [Figure 2]. This area of superior limbus was also associated with limited peripheral corneal neovascularization. Because of the evolving clinical appearance and the frequency and severity of her symptoms, excisional biopsy of the left superior conjunctiva was performed.

**Figure 1a:**
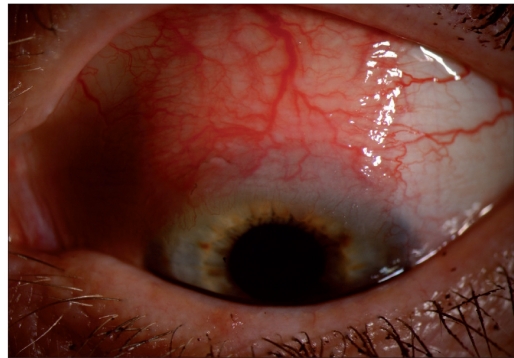
Superior conjunctival lesion with thickening, injection and prominent episcleral vessels

**Figure 1b:**
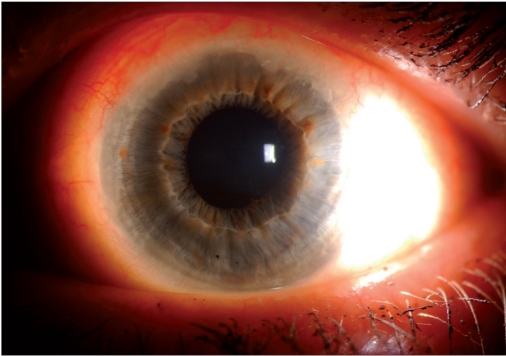
Close up view of the superior limbus highlighting peripheral neovascularization and limbal leukoplakia

**Figure 2:**
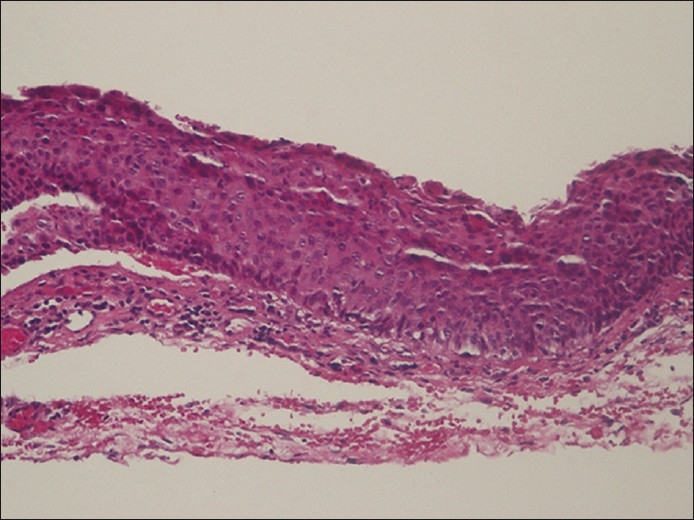
Pathology showing acanthotic, nonkeratinized epithelial layer. Dysplasia is noted 2/3 of the way through the specimen. Artificial separations are noted in the tissue from processing. The basement membrane is intact. Chronic inflammation is noted in the substantia propria, which can be seen in a chronically irritatedconjuntival lesion (H and E, ×200)

**Figure 3:**
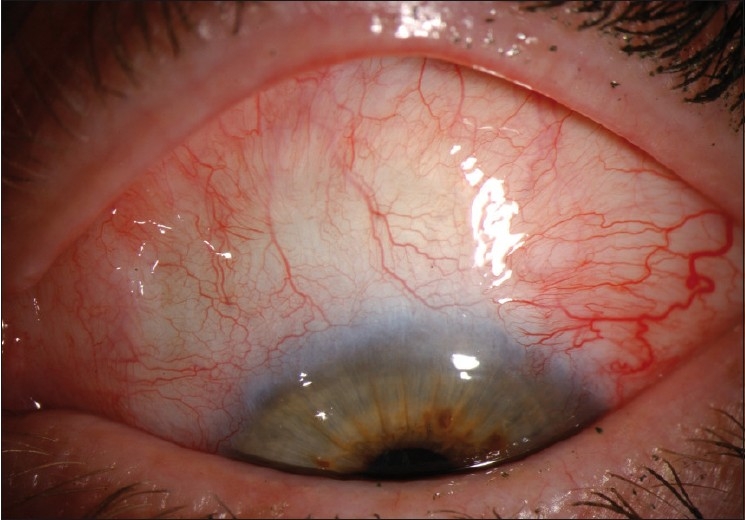
Slit lamp photograph showing resolution of the ocular surface squamous neoplasia

Pathologic examination with standard hematoxylin and eosin stains revealed diffuse thickening of the epithelium, with focal areas of dyskeratosis, atypia, pleomorphism, prominent nucleoli and clumped chromatin [Figure 3]. These changes extended to 50–80% of the epithelial thickness and there was no penetration of the epithelial basement membrane. A diagnosis of OSSN of the conjunctiva was made and the patient was started on mitomycin C 0.02% three-times a day for 3 weeks. On follow-up, the superior corneal epithelium showed persistent leukoplakia and treatment was changed to interferon alfa 2b (IFNα2b) eyedrops (2 million IU/ml) four-times daily, with resolution of clinical findings over 2 months.

## DISCUSSION

SLK is characterized by a fine papillary reaction on the superior tarsal conjunctiva, injection and thickening of the superior bulbar conjunctiva, and superior corneal filamentary keratopathy.^6^ The pathogenesis of this condition has not been well understood, but it is believed to result from superior bulbar conjunctiva laxity and concomitant inflammatory changes from mechanical soft tissue microtrauma.^7^ Factors causing superior bulbar conjunctiva laxity include thyroid eye disease and tight upper eyelids.

The location of OSSN is typically in the interpalpebral fissure,^8^ and the superior bulbar conjunctival location of our patient’s lesion is an atypical presentation. Such a presentation is more commonly associated with SLK. It is possible that the chronic irritation and mechanical trauma of SLK, which our patient presented with, induced a malignant transformation of the superior bulbar conjunctiva. The laxity induced by OSSN may have precipitated localized mechanical trauma and chronic inflammation. As a result, the initial clinical presentation in our patient imitated what is typically seen in SLK.

The transformation of the stratified epithelium into squamous carcinoma as a result of mechanical irritation has been described in the conjunctiva as a result of a poorly fitting prosthesis,^9^ in bladder cancer as a result of foley catheter irritation,^10^ and in colon cancer.^11^ Alternatively, the OSSN lesion may have been masquerading as SLK all along, and it was not until its corneal extension that it became evident.

OSSN presenting as cornea ulcer,1 chronic blepharoconjunctivitis,2 pterygium,3 necrotizing scleritis4 and sclerokeratitis5 have been reported in the literature and this is the first report of OSSN developing from or masquerading as SLK. In our case, the initial diagnosis of SLK was made based on the location of the lesion, classic slit-lamp appearance, clinical symptoms and patient’s associated past medical history of thyroid dysfunction.^12^ Because clinical features of conjunctival lesions may show similarities, it may be difficult to distinguish benign from invasive OSSN and from other common lesions such as pinguecula and pterygium. Our case highlights the importance of considering OSSN in refractory cases of conjunctival inflammation with limbal and corneal epithelial involvement.
